# Proteome Analysis of the Antiproliferative Activity of the Novel Chitooligosaccharide–Gallic Acid Conjugate against the SW620 Colon Cancer Cell Line

**DOI:** 10.3390/biomedicines11061683

**Published:** 2023-06-10

**Authors:** Jirakrit Saetang, Phutthipong Sukkapat, Ajay Mittal, Jakrawadee Julamanee, Wannakorn Khopanlert, Kajornkiat Maneechai, Rasool Abdul Nazeer, Surasak Sangkhathat, Soottawat Benjakul

**Affiliations:** 1 International Center of Excellence in Seafood Science and Innovation, Faculty of Agro-Industry, Prince of Songkla University, Hat Yai 90110, Songkhla, Thailand; jirakrit.s@psu.ac.th (J.S.); sukkapat001@gmail.com (P.S.); ajy.mittal@yahoo.com (A.M.); 2 Stem Cell Laboratory, Hematology Unit, Division of Internal Medicine, Faculty of Medicine, Prince of Songkla University, Hat Yai 90110, Songkhla, Thailand; jakrawadee.j@psu.ac.th (J.J.); wannakornknopanlert@gmail.com (W.K.); m.kajornkiat20@gmail.com (K.M.); 3 Biopharmaceuticals Lab, Department of Biotechnology, School of Bioengineering, SRM Institute of Science and Technology, Kattankulathur, Chennai 603203, Tamilnadu, India; nazeerr@srmist.edu.in; 4 Department of Surgery, Faculty of Medicine, Prince of Songkla University, Hat Yai 90110, Songkhla, Thailand; surasak.sa@psu.ac.th; 5 Translational Medicine Research Center, Faculty of Medicine, Prince of Songkla University, Hat Yai 90110, Songkhla, Thailand; 6 Department of Food and Nutrition, Kyung Hee University, Seoul 02447, Republic of Korea

**Keywords:** chitooligosaccharide, gallic acid, conjugate, anti-cancer activity, proteomics, LC-MS/MS

## Abstract

Chitooligosaccharide (COS) and gallic acid (GA) are natural compounds with anti-cancer properties, and their conjugate (COS–GA) has several biological activities. Herein, the anti-cancer activity of COS–GA in SW620 colon cancer cells was investigated. MTT assay was used to evaluate cell viability after treatment with 62.5, 122, and 250 µg/mL of COS, GA, and COS–GA for 24 and 48 h. The number of apoptotic cells was determined using flow cytometry. Proteomic analysis was used to explore the mechanisms of action of different compounds. COS–GA and GA showed a stronger anti-cancer effect than COS by reducing SW620 cell proliferation at 125 and 250 µg/mL within 24 h. Flow cytometry revealed 20% apoptosis after COS–GA treatment for 24 h. Thus, GA majorly contributed to the enhanced anti-cancer activity of COS via conjugation. Proteomic analysis revealed alterations in protein translation and DNA duplication in the COS group and the structural constituents of the cytoskeleton, intermediate filament organization, the mitochondrial nucleoid, and glycolytic processes in the COS–GA group. Anti-cancer-activity-related proteins were altered, including CLTA, HSPA9, HIST2H2BF, KRT18, HINT1, DSP, and VIM. Overall, the COS–GA conjugate can serve as a potential anti-cancer agent for the safe and effective treatment of colon cancer.

## 1. Introduction

Chitooligosaccharide (COS) is derived from chemical or enzymatic chitosan hydrolysis [1,2,3,4]. Generally, COS is defined by a degree of polymerization (DP) ranging from 2 to 10 monomers of glucosamine, a degree of deacetylation (DD) of more than 90%, and an average molecular weight of <3.9 kDa [5]. COS exhibits various bioactivities, including antioxidant [6], anti-obesity [7], anti-microbial [8], and anti-cancer activities [9,10], with non-toxic and non-allergenic properties [9,11]. The structure of COS contains three reactive functional groups, including amino groups, hydroxyl groups, and a glycosidic bond, which favor COS modification to enhance its bioactivity via chemical reactions [12]. Currently, modified forms of COS have been synthesized to produce biologically enhanced derivatives by linking the COS backbone with other bioactive constituents or chemical groups, such as polyphenols [2], carboxyl groups [13], aminoethyl groups [14], and sulfate groups [15].

Gallic acid (GA; 3,4,5-trihydroxybenzoic acid) is a bioactive polyphenolic compound in various plants, fruits, and herbal medicines [16]. GA comprises a benzene ring with three adjacent hydroxyl groups and one carboxylic group at the 1 position [16]. GA exhibits strong antioxidant activity [16,17] and possesses anti-cancer, anti-inflammatory, anti-microbial, and anti-fungal properties [18]. Therefore, the biological and pharmacological properties of GA have attracted the attention of researchers by attempting to improve its activity by combining it with other bioactive ingredients. The COS–GA conjugate demonstrates various pharmacological functions. The COS–GA conjugate displays a higher ability to suppress the proliferation of human gastric cancer cell lines than COS [19]. Furthermore, COS–GA inhibits allergic reactions [20]. COS–GA also outperforms COS in free radical scavenging in mouse macrophage RAW264.7 cells [21]. Although the anti-cancer activity of COS–GA has been elucidated, its suppressive ability against colon cancer, especially in the metastatic form, has not been evaluated.

Colon cancer is a leading cause of cancer-related deaths worldwide, ranking third in incidence and second in mortality rates among men and women [22]. In Thailand, colon cancer was the fourth-most commonly diagnosed malignancy in 2020, accounting for 11.1% of all new cancer cases [22]. Surgery, chemotherapy, and targeted therapy are the conventional treatments for colon cancer [23]. However, the occasional toxicity and ineffectiveness of therapeutic agents in patients with multiple metastases consequently decrease the overall survival of the patients to a few months [24,25]. Moreover, surgery is not always suitable for older patients at risk for postoperative complications and mortality [26]. Thus, novel therapeutic agents, particularly those derived from natural sources, must be developed to overcome these limitations for cancer treatment.

High-throughput methods, such as proteomic analysis, are considered powerful tools for understanding the mechanisms underlying cellular biological processes, especially in oncology research. Various apoptotic induction pathways have been identified using proteomic analysis. Endoplasmic reticulum stress and reactive oxygen species (ROS)-mediated pathways have been proposed to trigger apoptosis in human cervical cancer HeLa cells treated with betulinic acid using 2D gel electrophoresis and mass spectrometry [27]. Furthermore, molecular targets associated with the anti-cancer activity of curcumin were identified using a curcumin probe (Cur-P) combined with liquid chromatography–tandem mass spectrometry (LC-MS/MS) analysis. Over 100 proteins have been identified in the HCT116 colon cancer cell line as curcumin-binding proteins involved in various metabolic pathways and cellular processes [28]. In addition, activation of the stress response, cell cycle, and DNA repair pathways promoted apoptosis in a triple-negative MDA-MB-231 breast cancer cell line treated with *Lippia origanoides* extracts using label-free quantitative proteomic analysis [29].

Since the proteomic approach has been demonstrated to be a high-impact method for identifying differentially expressed proteins (DEPs) of the components in biological processes, this study used this tool to characterize the proteins associated with apoptosis in the highly metastatic colon cancer cell line, SW620, after treatment with COS and COS–GA. Candidate proteins and biological pathways were elucidated, highlighting the important mechanistic regulation of COS–GA in anti-cancer activity.

## 2. Materials and Methods

### 2.1. COS and COS–GA Preparation

COS and COS–GA were prepared as previously described [2]. COS was prepared by hydrolyzing shrimp chitosan by mixing 1% (*w*/*v*) chitosan with 2% (*v*/*v*) acetic acid (Merck KGaA, Darmstadt, Germany) for 16–18 h at 25 °C, followed by adjusting the pH to 5.0 using 6 M NaOH solution (Merck KGaA, Darmstadt, Germany). Subsequently, 2 mL of ascorbic acid (AsA)/H_2_O_2_ redox pair solution was added to the chitosan solution (100 mL) and shaken at 60 °C for 2 h before pH adjustment to 7.0 using 6 M NaOH. The supernatant containing COS was collected using centrifugation at 10,000× *g* for 15 min at 25 °C (Eppendorf 5424R, Hamburg, Germany). COS–GA was prepared by adjusting the pH of the COS solution to 5.0 using acetic acid and mixing it with AsA/H_2_O_2_ redox pair solution. The mixture was incubated at 25 °C for 1 h with continuous stirring using a magnetic stirrer. GA (Sigma-Aldrich, Darmstadt, Germany) was added to the solution at a final concentration of 0.4% (*w*/*v*). The mixture was then incubated at 25 °C for 24 h in the dark. All solutions were dialyzed against 20 volumes of distilled water for 24 h at 4 °C using a 500 Da cut-off dialysis bag and lyophilized (Scanvac, Lynge, Denmark). The obtained powder was placed in a zip-lock bag and stored at −40 °C in a freezer until use.

### 2.2. Cell Culture and MTT Assay

SW620 cells were obtained from the American Type Culture Collection (ATCC; Manassas, VA, USA). The cells were maintained in Leibovitz’s L-15 Medium (Gibco, Grand Island, NY, USA) containing 10% fetal bovine serum (Gibco) and 1% antibiotic–antimycotic (Gibco) at 37 °C in a 5% CO_2_ incubator (BINDER GmbH, Tuttlingen, Germany). For MTT assay, the cell proliferation kit I (Roche Diagnostics, Basel, Switzerland) was used according to the manufacturer’s instructions. Briefly, SW620 cells were seeded in a 96-well plate at 1 × 10^4^ cells/well and incubated at 37 °C in a CO_2_ incubator. After 24 h of incubation, the cells were treated with COS, GAand COS–GA at different concentrations (62.5, 122, and 250 µg/mL) for 24 and 48 h. MTT solution (10 µL) was added to each well, and the cells were continuously incubated for 4 h in a CO_2_ incubator before adding 100 µL of solubilization solution. After 16–18 h of incubation, the absorbance of each well was measured at 570 nm using a FLUOstar^®^ Omega microplate reader (BMG Labtech, Ortenberg, Germany).

### 2.3. Flow Cytometry of Apoptosis Analysis

SW620 cells were counted and seeded in a 6-well plate at 2 × 10^5^ cells/well in 2 mL of the completed medium/well before incubation at 37 °C in a CO_2_ incubator for 24 h. The cells were subsequently treated with COS, GA and COS–GA at varying concentrations (62.5, 122, and 250 µg/mL) for 24 and 48 h. Apoptotic cells were then quantified by staining the cells with the FITC Annexin V apoptosis detection kit with propidium iodide (PI; Biolegend, San Diego, CA, USA) according to the manufacturer’s instructions. Briefly, cells were harvested, washed twice with cell-staining buffer (0.5% bovine serum albumin in phosphate-buffered saline), and resuspended in Annexin V Binding Buffer. Thereafter, 100 µL of the cell suspension was mixed with 5 µL of FITC Annexin V dye and 10 µL of PI solution before incubating at 25 °C for 15 min. The volume of the cell suspension was then adjusted by adding 400 µL of Annexin V Binding Buffer, and the sample was subjected to flow cytometry analysis using FACSAria II (BD Biosciences, San Jose, CA, USA).

### 2.4. LC-MS/MS Analysis

Total protein was extracted and determined using the Lowry method. For peptide preparation, 10 µg of protein was separated using SDS-PAGE before dehydration with acetonitrile. Proteins were treated with 10 mM DTT (Merck KGaA, Darmstadt, Germany) at 56 °C for 1 h to reduce the sulfhydryl group and incubated with 100 mM iodoacetamide (Sigma-Aldrich, St. Louis, MO, USA) in the dark for 45 min for alkylation. The gel was then dehydrated by incubating in acetonitrile for 5 min before digestion with trypsin (Promega, Mannheim, Germany) at 37 °C for 16–18 h. The tryptic peptides were then subjected to dimethyl labeling before loading to the EASY-Spray™ C18, 75 cm × 75 μm column (Thermo Fisher Scientific, Waltham, MA, USA) and separated with the gradient using mobile phase A (0.1% formic acid in LC-MS-grade water) and mobile phase B (0.1% formic acid in LC-MS-grade acetonitrile) for 90 min with a flow rate of 300 nL/min. Cellular proteins were analyzed using LC-MS/MS operated on the EASY-nLC 1000 liquid chromatography (Thermo Fisher Scientific) and the Q exactive™ plus hybrid quadrupole-orbitrap™ mass spectrometer (Thermo Fisher Scientific). Proteins were then identified based on their spectra using Proteome Discoverer 2.1 software (Thermo Fisher Scientific) and the human UniProt database (https://www.uniprot.org/, accessed on 20 October 2022).

### 2.5. LC-MS/MS Data Analysis

Statistical analysis was performed, and LC-MS/MS data were filtered using Perseus software v.1.6.0.7 [30]. Term enrichment of Gene Ontology (GO) and biological pathways were identified using GO analysis tools (http://geneontology.org/, accessed on 5 October 2022) and Kyoto Encyclopedia of Genes and Genomes (KEGG) pathway analysis (https://www.genome.jp/kegg/, accessed on 21 October 2022). A heat map was constructed using GraphPad Prism version 9 (San Diego, CA, USA) based on the abundance scale.

### 2.6. Statistical Analysis

All statistical analyses were performed using SPSS version 26 (IBM, Armonk, NY, USA). Analysis of variance (ANOVA) followed by Tukey’s test was used for comparison. Student’s *t*-test was performed for pair comparison. Results with a *p*-value of <0.05 were considered statistically significant. All experiments were performed in triplicate.

## 3. Results

### 3.1. Cytotoxicity of COS, Gallic Acid, and COS–GA on SW620 Cells

**Figure 1:**
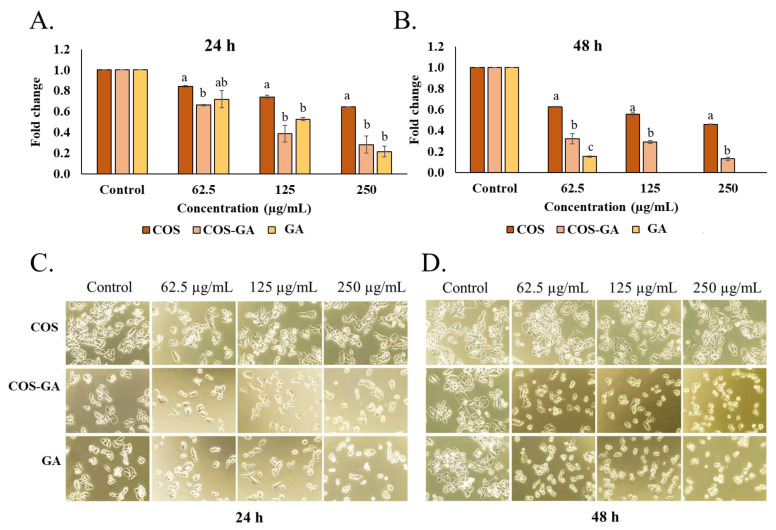
Cell viability after treatment with COS and COS–GA. MTT assay of SW620 cells after COS, GA, and COS–GA treatment at different concentrations for 24 h (**A**) and 48 h (**B**). SW620 cell morphology after COS, GA, and COS–GA treatment for 24 h (**C**) and 48 h (**D**). Data are shown as means ± standard deviations from triplicate experiments. Different lowercase letters on the bars within the same concentration denote statistically significant differences (*p* < 0.05).

**Figure 2:**
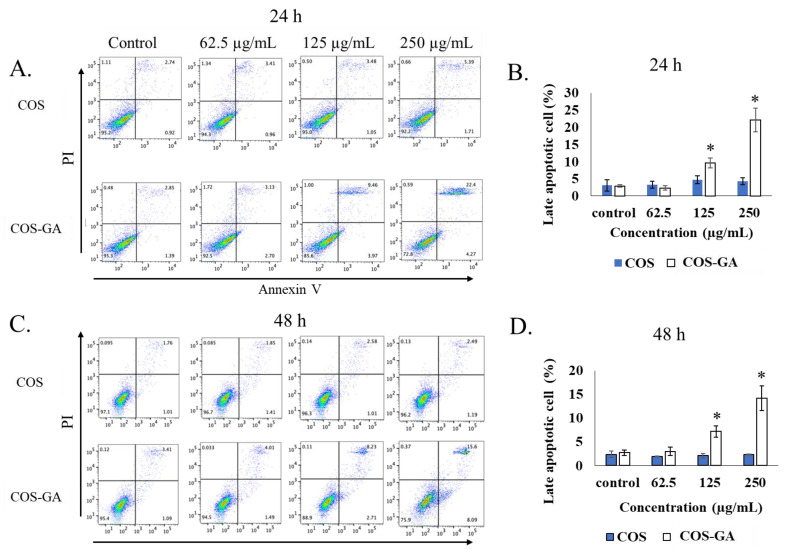
Flow cytometry of apoptotic cells. (**A**) The representative dot plot from flow cytometry analysis of SW620 cells after treatment with COS and COS–GA at different concentrations for 24 h (**A**) and 48 h (**C**). The percentage of late apoptotic cells after treatment with COS and COS–GA at different concentrations for 24 h (**B**) and 48 h (**D**). Data are shown as means ± standard deviations from triplicate experiments, analyzed with one-way ANOVA followed by post hoc Tukey’s tests. * *p* < 0.05 indicates statistically significant differences.

To evaluate the anti-cancer activity of COS, GA, and COS–GA, SW620 cells were selected as an in vitro model for cytotoxicity testing. At 24 h, more than 50% of cell viability was observed at all COS concentrations used in comparison with the control (Figure 1A). However, approximately 50% reduction in cell viability was observed at 250 µg/mL after 48 h of COS treatment (Figure 1B). GA showed strong cytotoxicity (cell viability < 50%) toward SW620 cells at 250 µg/mL after 24 h (Figure 1A), while its effect toward the cells was more pronounced even at the concentration of 62.5 µg/mL after 48 h (Figure 2B). Nonetheless, no data of GA-treated samples were reported at the concentrations of 125 and 250 µg/mL, due to the disturbing color of GA (Figure 2B). GA at high concentrations more likely underwent oxidation to quinone, causing the darker color, which had an interfering effect on the assay. COS–GA affected the viability of SW620 cells treated at 125 and 250 µg/mL for both 24 and 48 h (Figure 1A,B). A decrease of approximately 20–50% in live cells was observed at 125 µg/mL of COS–GA, whereas more than 50% of SW620 cells lost their viability when the COS–GA concentration increased to 250 µg/mL. Overall, GA showed a higher cytotoxicity effect on the cells than COS but had a similar impact as the COS–GA conjugate when the same concentration was used. Thus, GA conjugated with COS mainly contributed to the augmented cytotoxicity of the resulting conjugate.

When the SW620 cell morphology was examined after COS, GA, and COS–GA treatment, the original shape with an evident cell border and close contact was observed in untreated SW620 cells at both 24 and 48 h (Figure 1C,D). However, a spherical shape and a cell–cell fusion cluster with cell detachment from the cell container bottom were observed when SW620 cells were treated with GA and COS–GA for both 24 and 48 h. With the same incubation time and dose, COS showed a lower effect on the SW620 cell morphology and behavior than COS–GA or GA. This result reconfirmed the profound role in the enhancement of the anti-cancer activity of COS via conjugation with GA.

### 3.2. Apoptotic Induction Effect of COS and COS–GA on SW620 Cells

Since it was evident that GA improved the anti-cancer properties of COS, only COS was used as a control for the rest of the experiments. To evaluate the apoptosis induction effect of COS and COS–GA on SW620 cells, FITC Annexin V staining and flow cytometry were used to monitor the apoptotic behavior of the treated cells. Interestingly, COS–GA treatment induced apoptosis dose dependently, whereas no significant apoptotic cell death was observed when the cells were incubated with COS (Figure 2). COS–GA caused late apoptosis in SW620 cells up to 10% and 22% when treated at 125 and 250 µg/mL, respectively, for 24 h (Figure 2A,B). In particular, a significant difference in apoptotic induction activity was observed between COS and COS–GA in the dot plot analysis. The late apoptotic cells indicated by the double positive staining of PI and Annexin V (top right quadrant) increased from approximately 5% when treated with 250 µg/mL of COS to approximately 22% when treated with 250 µg/mL of COS–GA for 24 h (Figure 2A). A similar trend was observed when the cells were treated for 48 h (Figure 2C). However, the dot plot analysis showed that the proportions of late apoptotic cells were approximately 8% and 15% in SW620 cells treated with 125 and 250 µg/mL of COS–GA for 48 h, respectively (Figure 2C,D). Although a small proportion of the cells appeared to increase in the late apoptotic quadrant after 48 h of 250 µg/mL COS treatment, apoptosis was not significantly induced in SW620 cells.

### 3.3. Proteomic Profiling of COS- and COS–GA-Treated SW620 Cells

**Figure 3:**
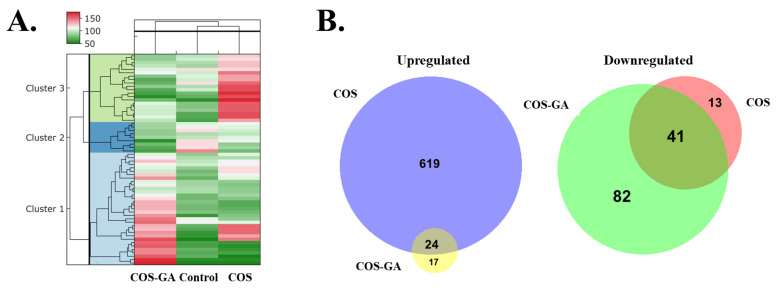
LC-MS/MS analysis of differentially expressed proteins of SW620 cells treated with COS, COS–GA, and the control for 24 h. Data are represented as heat map protein profiles (**A**) and Venn diagrams comparing the number of upregulated proteins and downregulated proteins between COS and COS–GA groups ((**B**) left and right panels, respectively).

To investigate the molecular characteristics of COS- and COS–GA-treated SW620 cells, the effective dose of 250 µg/mL was used in comparison with the untreated control, followed by a proteome profile study using LC-MS/MS-based quantitative proteomics analysis. The protein abundance in each group is shown using a heat map (Figure 3A) and Venn diagrams (Figure 3B). Over 700 valid proteins were identified in all groups (Supplementary Material Table S1). Unsupervised clustering analysis of the quantified proteins revealed three clusters showing differences in protein expression patterns between the groups. Cluster 1 comprised upregulated proteins found only in the COS–GA group compared to the control group, whereas Cluster 3 included less abundant proteins in the COS–GA group but increased abundance in the COS group compared to the control group. Proteins without significant changes were categorized into Cluster 2 in the heat map with a small proportion. To compare the molecular roles of COS and COS–GA in SW620 cells, the high-abundance and low-abundance proteins of the two groups were analyzed and expressed in Venn diagrams. In total, 24 proteins were upregulated and 41 were downregulated in both groups (Figure 3B).

### 3.4. Functional Annotation of Altered Proteins in COS- and COS–GA-Treated SW620 Cells

**Figure 4:**
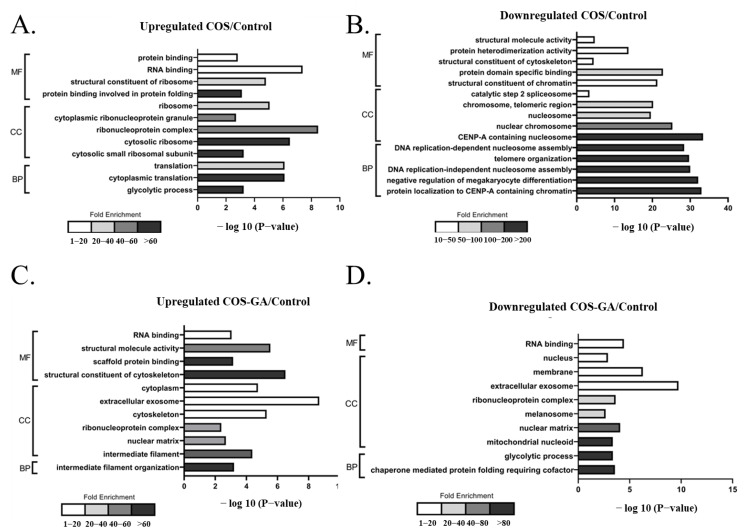
Analysis of the functional pathways of the differentially expressed proteins in COS- and COS–GA-treated SW620 cells. Pathways upregulated (**A**) and downregulated (**B**) in the COS treatment group compared to the control group. Pathways upregulated (**C**) and downregulated (**D**) in the COS–GA treatment group compared to the control group.

To determine the molecular pathways involved in the COS and COS–GA response to SW620 cells, functional analysis related to molecular function (MF), cellular components (CC), and biological processes (BP) was performed. The proteins, both upregulated and downregulated in both treatments, were annotated using GO enrichment analysis, which provided the most relevant GO pathways. The most abundant proteins with the most statistical significance in the COS group were proteins involved in protein folding, the cytosolic ribosome, and cytoplasmic translation in the MF, CC, and BP groups, respectively (Figure 4A). The downregulated proteins with more than 200-fold enrichment were related to the organization and assembly of nucleosomes and telomeres in the COS-treated group, with a significant −log *p*-value of −25 to −35 (Figure 4B). GO annotations upregulated in the COS–GA group included structural constituents of the cytoskeleton, intermediate filaments, and intermediate filament organization for the MF, CC, and BP groups, respectively (Figure 4C). For downregulated pathways, the highest fold enrichment was found for the mitochondrial nucleoid, glycolytic process, and chaperone-mediated protein folding requiring cofactors (Figure 4D).

### 3.5. Candidate Proteins Related to the Anti-Cancer Mechanism Based on a Literature Search

To identify proteins that may play a role in cancer cell viability, the significantly upregulated and downregulated proteins in COS and COS–GA groups with changes of more than two folds were subjected to a scientific literature search using the keywords “protein name”, “cancer”, and “apoptosis” compared to those of the control group. Proteins reported in ≥2 articles for their effect on cancer biology and expression trends were consistent with the current findings as the key proteins for SW620 inhibition. Interestingly, only one protein (CLTA) was associated with anti-cancer activity in the COS group in an increased expression manner (Table 1). However, six proteins influenced cancer cell survival in the COS–GA group in previous studies, with downregulated HSPA9 and HIST2H2BF and upregulated KRT18, HINT1, DSP, and VIM proteins. Among other viability-associated proteins, KRT18 showed the highest levels of expression, with an average log2 ratio of 2.71 in the COS–GA/control group, whereas HSPA9 was the most downregulated protein, with a −2.02 average log2 ratio. CLTA upregulation found in COS-treated SW620 cells demonstrated the lowest upregulation levels, with a 1.21 average log2 ratio compared to the control group.

## 4. Discussion

COS is a chitosan-derived molecule with a low molecular weight and high water solubility [46]. Several biological activities of COS have been reported, including anti-cancer capability [47]; however, this effect is dependent on the COS size. COS containing 3–5 monomers showed stronger anti-cancer activity than high-molecular-weight chitosan and hetero-COS (COS with 6–15 monomers) when tested in human PC3 (prostate), A549 (lung), and HepG2 (liver) cancer cells [48]. Over 75% of all cancer cell types were inhibited when 50 µg/mL of COS was used. In this study, COS exhibited a negligible effect on cancer cell inhibition, even when 250 µg/mL was used. This may be due to the different characteristics of the COS samples. COS had an average molecular weight of 0.7 kDa, with 2–8 DP and 91% DD [2]. This is supported by another previous study showing that higher doses (1–5 mg/mL) and longer treatment times (72 h) are required for anti-cancer activity when 1–2 kDa of COS is used [49].

Interestingly, COS–GA conjugation enhanced the cancer inhibitory effect by promoting apoptosis at 125 and 250 µg/mL. The cytotoxicity of COS–GA was higher than that of COS at the same concentration, but no significant difference was observed between GA and COS–GA in the MTT assay. Since the number of GA molecules in COS–GA was more likely lower than that in purified GA based on the same weight, it implied that the anti-cancer activity found in COS–GA was from the synergy between COS and GA. This augmented anti-cancer phenomenon may be caused by a combination of the anti-cancer mechanisms of both GA and COS. GA is an anti-tumorigenic agent with antioxidant and anti-inflammatory activities [50]. The anticarcinogenic mechanisms of GA may induce ROS-dependent mitochondrial apoptosis [51] and cell lipid peroxidation [52]. In contrast, the cationic charge of COS, which has been postulated to facilitate the binding of its molecule to glycoproteins and charged residues on the tumor cell membrane, might lead to cell permeability loss [53]. YKL-40 glycoprotein, which is overexpressed on the cell membranes of various cancers, is a COS-binding target [47]. YKL-40 also functions as an anti-apoptotic protein and an angiogenic factor that promotes cancer progression [54,55] and increases in patients with colorectal cancer [56]. Therefore, the COS in COS–GA may selectively bind to colon cancer cells. In addition to cell membrane disruption mechanism, COS might function as a carrier to facilitate the exposure of cancer cells to GA, resulting in an increase in the number of apoptotic cells compared to COS treatment alone. This result is consistent with the findings of Ryu et al. [19], who demonstrated that GA-grafted COS augments the anti-cancer activity of COS when tested in AGS human gastric cancer cells.

In this study, a proteomic approach was used to identify the proteins involved in the responsive mechanism of SW620 cells to COS and COS–GA compared to the control group. Different protein profiles in each treated group implied the different pathways in COS- and COS–GA-treated SW620 cells. No significant apoptosis was found in the COS-treated cells. COS likely altered the processes of the ribonucleoprotein complex, cytoplasmic translation, and nucleosome assembly, all of which are related to the alteration of protein translation and DNA duplication. It has been suggested that a longer treatment period and a higher COS dose are required to significantly inhibit cancer cell growth. In the COS–GA group, DEPs with more than two-fold changes indicated molecular pathways involved in some biological functions, including intermediate filament organization and structural constituents of the cytoskeleton, which increased in COS–GA-treated cells. Interestingly, intermediate filament reorganization is normally associated with the formation of an apoptotic microtubule network during the execution phase of the apoptotic pathway [57]. Our study reported similar results of the increasing apoptotic trend in this group. Furthermore, the glycolytic process was downregulated in COS–GA-treated SW620 cells, which is related to caspase-dependent inhibition of glycolysis during apoptosis [58]. Moreover, the decrease in pathways associated with both the nuclear matrix and the mitochondrial nucleoid has been considered a scenario that could be observed in apoptotic cells [59,60,61].

Many proteins in SW620 cells were differentially expressed after COS–GA treatment, and some were associated with cancer cell viability. For example, the HSPA9 protein, which was significantly downregulated by approximately four folds in the COS–GA group, exhibited anti-apoptotic effects on tumor cells by decreasing the levels of TP53 tumor suppressor protein [32]. However, the depletion of this protein alters the polarization of the mitochondrial membrane, changes cell metabolism and environmental acidification, and results in higher oxidative stress, leading to cell death [34]. Another significantly downregulated protein in COS–GA-treated SW620 cells, HIST2H2BF, is a core component of the nucleosome and plays an important role in colon cancer stemness, migration, invasion, and metastasis [35].

Among the upregulated proteins, keratin 18 expression significantly increased in the COS–GA group. Keratin 18 controls the expression of apoptotic proteins, such as FAS, FADD, and caspase 8 [38]. Increasing keratin 18 levels promote apoptosis by inducing pro-apoptotic protein expression. In addition, keratin 18 enhances the expression of cell adhesion molecules and reduces cancer aggressiveness in vivo [37]. HINT1, a highly upregulated protein in the COS–GA group, suppresses tumors by inhibiting the expression of the cell cycle regulatory protein, cyclin D1, promoting the expression of pro-apoptotic factors, such as Bax and TP53, and alleviating metastatic capability by altering the girdin and AKT signaling pathways [39,40,41]. Although vimentin promotes cancer metastasis, its antitumorigenic role has been recently reported. Silencing the vimentin protein in cancer cells activates AKT and β-catenin signaling and prevents AMPK-mediated autophagy [44]. The Wnt/β-catenin signaling cascade is the initiating driver for colon cancer tumorigenesis [62]. Vimentin plays an important role in suppressing intestinal inflammation involved in colon cancer development [45].

Although the molecular mechanism of COS–GA in cancer inhibition was not thoroughly investigated in this study, the proteomic approach revealed some biological processes associated with apoptosis. Nevertheless, previous studies with similar mechanisms of COS, GA, and COS–GA as in this study have been performed. For example, GA inhibits colon cancer cell growth by limiting glycolysis, inducing apoptosis, and promoting cell cycle arrest [63]. COS treatment reduces cell proliferation and induces cell apoptosis through the TP53/mTOR signaling pathway [9]. These findings indicate that the combination of COS and GA enhances the cells’ inhibitory activity against cancer progression.

## 5. Conclusions

COS–GA showed a superior anti-cancer effect on SW620 cells via the induction of cancer cell apoptosis. Alterations in the levels of some proteins in the COS–GA group, such as HSPA9, HIST2H2BF, keratin 18, HINT1, and vimentin, were proposed as anti-cancer mechanisms in this study. Our findings may help in the development of alternative treatments for colon cancer, although the underlying mechanisms require further investigation. Nevertheless, Western blotting or RT-qPCR studies should be conducted to validate the key up-/downregulated protein candidates.

## Figures and Tables

**Table 1:** List of literature-based viability-associated proteins found in SW620 cells.

Accession/Expression	Description	Gene Name	Average log2 Ratio	Oncogenic Role	Ref.
**COS**					
**Upregulation**					
P09496-2	Isoform non-brain of clathrin light chain A	*CLTA*	1.21	Anti-tumorigenic	[31]
**COS-G**					
**Downregulation**					
P38646	Stress-70 protein, mitochondrial	*HSPA9*	−2.02	Pro-tumorigenic	[32,33,34]
Q5QNW6-2	Isoform 2 of histone H2B type 2-F	*HIST2H2BF*	−1.93	Pro-tumorigenic	[35,36]
**Upregulation**					
P05783	Keratin, type I cytoskeletal 18	*KRT18*	2.71	Anti-tumorigenic	[37,38]
P49773	Histidine triad nucleotide-binding protein 1	*HINT1*	1.90	Anti-tumorigenic	[39,40,41]
P15924	Desmoplakin	*DSP*	1.35	Anti-tumorigenic	[42,43]
P08670	Vimentin	*VIM*	1.25	Anti-tumorigenic	[44,45]

## Data Availability

The data presented in this study are available upon request from the corresponding author.

## Supplementary Materials

The following supporting information can be downloaded at https://www.mdpi.com/article/10.3390/biomedicines11061683/s1: Table S1: Proteins identified by LC-MS/MS; Table S2: Protein names and abundance ratio of altered proteins.

## References

[1] Singh A., Benjakul S., Huda N., Xu C., Wu P. (2020). Preparation and characterization of squid pen chitooligosaccharide–epigallocatechin gallate conjugates and their antioxidant and antimicrobial activities. RSC Adv..

[2] Mittal A., Singh A., Zhang B., Visessanguan W., Benjakul S. (2022). Chitooligosaccharide conjugates prepared using several phenolic compounds via ascorbic acid/H_2_O_2_ free radical grafting: Characteristics, antioxidant, antidiabetic, and antimicrobial activities. Foods.

[3] Nirmal N.P., Santivarangkna C., Rajput M.S., Benjakul S. (2020). Trends in Shrimp Processing Waste Utilization: An Industrial Prospective. Trends Food Sci. Technol..

[4] Hosney A., Ullah S., Barčauskaitė K. (2022). A review of the chemical extraction of chitosan from shrimp wastes and prediction of factors affecting chitosan yield by using an artificial neural network. Mar. Drugs.

[5] Liaqat F., Eltem R. (2018). Chitooligosaccharides and their biological activities: A comprehensive review. Carbohydr. Polym..

[6] Yang F., Luan B., Sun Z., Yang C., Yu Z., Li X. (2017). Application of chitooligosaccharides as antioxidants in beer to improve the flavour stability by protecting against beer staling during storage. Biotechnol. Lett..

[7] Jin Q., Yu H., Wang X., Li K., Li P. (2017). Effect of the molecular weight of water-soluble chitosan on its fat-/cholesterol-binding capacities and inhibitory activities to pancreatic lipase. PeerJ.

[8] Laokuldilok T., Potivas T., Kanha N., Surawang S., Seesuriyachan P., Wangtueai S., Phimolsiripol Y., Regenstein J.M. (2017). Physicochemical, antioxidant, and antimicrobial properties of chitooligosaccharides produced using three different enzyme treatments. Food Biosci..

[9] Pan Z., Cheng D., Wei X., Li S., Guo H., Yang Q. (2021). Chitooligosaccharides inhibit tumor progression and induce autophagy through the activation of the P53/MTOR pathway in osteosarcoma. Carbohydr. Polym..

[10] Mattaveewong T., Wongkrasant P., Chanchai S., Pichyangkura R., Chatsudthipong V., Muanprasat C. (2016). Chitosan oligosaccharide suppresses tumor progression in a mouse model of colitis-associated colorectal cancer through AMPK activation and suppression of NF-ΚB and MTOR signaling. Carbohydr. Polym..

[11] Ngo D.H., Vo T.S., Ngo D.N., Kang K.H., Je J.Y., Pham H.N.D., Byun H.G., Kim S.K. (2015). Biological effects of chitosan and its derivatives. Food Hydrocoll..

[12] Guan G., Azad M.A.K., Lin Y., Kim S.W., Tian Y., Liu G., Wang H. (2019). Biological effects and applications of chitosan and chito-oligosaccharides. Front Physiol..

[13] Rajapakse N., Kim M.M., Mendis E., Huang R., Kim S.K. (2006). Carboxylated chitooligosaccharides (CCOS) inhibit MMP-9 expression in human fibrosarcoma cells via down-regulation of AP-1. Biochim. Biophys. Acta.

[14] Hong S., Ngo D.N., Kim M.M. (2016). Inhibitory effect of aminoethyl-chitooligosaccharides on invasion of human fibrosarcoma cells. Environ. Toxicol. Pharmacol..

[15] Artan M., Karadeniz F., Karagozlu M.Z., Kim M.M., Kim S.K. (2010). Anti-HIV-1 activity of low molecular weight sulfated chitooligosaccharides. Carbohydr. Res..

[16] Bai J., Zhang Y., Tang C., Hou Y., Ai X., Chen X., Zhang Y., Wang X., Meng X. (2021). Gallic acid: Pharmacological activities and molecular mechanisms involved in inflammation-related diseases. Biomed. Pharmacother..

[17] Phonsatta N., Deetae P., Luangpituksa P., Grajeda-Iglesias C., Figueroa-Espinoza M.C., Le Comte J., Villeneuve P., Decker E.A., Visessanguan W., Panya A. (2017). Comparison of antioxidant evaluation assays for investigating antioxidative activity of gallic acid and its alkyl esters in different food matrices. J. Agric. Food Chem..

[18] Wianowska D., Olszowy-Tomczyk M. (2023). A concise profile of gallic acid—From its natural sources through biological properties and chemical methods of determination. Molecules.

[19] Ryu B., Kim S.Y., Vo T.S., Kim W.S., Kim D.G., Kim S.K. (2017). Characterization of the in vitro effects of gallic acid-grafted-chitooligosaccharides in the suppression of AGS human gastric cancer cell proliferation. RSC Adv..

[20] Vo T.S., Ngo D.H., Kim S.K. (2012). Gallic acid-grafted chitooligosaccharides suppress antigen-induced allergic reactions in RBL-2H3 mast cells. Eur. J. Pharm. Sci..

[21] Ngo D.H., Qian Z.J., Vo T.S., Ryu B., Ngo D.N., Kim S.K. (2011). Antioxidant activity of gallate-chitooligosaccharides in mouse macrophage RAW264.7 cells. Carbohydr. Polym..

[22] Sung H., Ferlay J., Siegel R.L., Laversanne M., Soerjomataram I., Jemal A., Bray F. (2021). Global Cancer Statistics 2020: GLOBOCAN estimates of incidence and mortality worldwide for 36 cancers in 185 countries. CA Cancer J. Clin..

[23] Cao C., Yan T.D., Black D., Morris D.L. (2009). A systematic review and meta-analysis of cytoreductive surgery with perioperative intraperitoneal chemotherapy for peritoneal carcinomatosis of colorectal origin. Ann. Surg. Oncol..

[24] Lemoine L., Sugarbaker P., Van der Speeten K. (2016). Pathophysiology of colorectal peritoneal carcinomatosis: Role of the peritoneum. World J. Gastroenterol..

[25] Sugarbaker P.H. (2016). Improving oncologic outcomes for colorectal cancer at high risk for local-regional recurrence with novel surgical techniques. Expert Rev. Gastroenterol. Hepatol..

[26] Groza D., Gehrig S., Kudela P., Holcmann M., Pirker C., Dinhof C., Schueffl H.H., Sramko M., Hoebart J., Alioglu F. (2018). Bacterial ghosts as adjuvant to oxaliplatin chemotherapy in colorectal carcinomatosis. Oncoimmunology.

[27] Xu T., Pang Q., Zhou D., Zhang A., Luo S., Wang Y., Yan X. (2014). Proteomic investigation into betulinic acid-induced apoptosis of human cervical cancer HeLa cells. PLoS ONE.

[28] Wang J., Zhang J., Zhang C.-J., Wong Y.K., Lim T.K., Hua Z.C., Liu B., Tannenbaum S.R., Shen H.M., Lin Q. (2016). In situ proteomic profiling of curcumin targets in HCT116 colon cancer cell line. Sci. Rep..

[29] Raman V., Aryal U.K., Hedrick V., Ferreira R.M., Fuentes Lorenzo J.L., Stashenko E.E., Levy M., Levy M.M., Camarillo I.G. (2018). Proteomic analysis reveals that an extract of the plant *Lippia origanoides* suppresses mitochondrial metabolism in triple-negative breast cancer cells. J. Proteome. Res..

[30] Tyanova S., Temu T., Cox J. (2016). The MaxQuant computational platform for mass spectrometry-based shotgun proteomics. Nat. Protoc..

[31] Rivera M., Ramos Y., Rodríguez-Valentín M., López-Acevedo S., Cubano L.A., Zou J., Zhang Q., Wang G., Boukli N.M. (2017). Targeting multiple pro-apoptotic signaling pathways with curcumin in prostate cancer cells. PLoS ONE.

[32] Liu T., Krysiak K., Shirai C.L., Kim S., Shao J., Ndonwi M., Walter M.J. (2017). Knockdown of HSPA9 induces TP53-dependent apoptosis in human hematopoietic progenitor cells. PLoS ONE.

[33] Peng C., Yang P., Cui Y., He M., Liang L., Di Y. (2013). HSPA9 overexpression inhibits apoptin-induced apoptosis in the HepG2 cell line. Oncol. Rep..

[34] Starenki D., Hong S.K., Lloyd R.V., Park J.I. (2015). Mortalin (GRP75/HSPA9) upregulation promotes survival and proliferation of medullary thyroid carcinoma cells. Oncogene.

[35] Qiu L., Yang X., Wu J., Huang C., Miao Y., Fu Z. (2021). HIST2H2BF potentiates the propagation of cancer stem cells via notch signaling to promote malignancy and liver metastasis in colorectal carcinoma. Front Oncol..

[36] Zeng Z., Lu J., Wu D., Zuo R., Li Y., Huang H., Yuan J., Hu Z. (2021). Poly(ADP-Ribose) glycohydrolase silencing-mediated H2B expression inhibits benzo(a)pyrene-induced carcinogenesis. Environ. Toxicol..

[37] Bühler H., Schaller G. (2005). Transfection of keratin 18 gene in human breast cancer cells causes induction of adhesion proteins and dramatic regression of malignancy In Vitro and In Vivo. Mol. Cancer Res..

[38] Cheng Y., Qin K., Huang N., Zhou Z., Xiong H., Zhao J., Zhang Y., Yu S. (2019). Cytokeratin 18 regulates the transcription and alternative splicing of apoptotic-related genes and pathways in HeLa cells. Oncol. Rep..

[39] Duan D.D., Xie H., Shi H.F., Huang W.W., Ding F., Hong J.K., Fan J.S., Hu S.Y., Wang Q.W., Zhou M.Q. (2020). Hint1 overexpression inhibits the cell cycle and induces cell apoptosis in human osteosarcoma cells. Onco. Targets Ther..

[40] Weiske J., Huber O. (2006). The histidine triad protein Hint1 triggers apoptosis independent of Its enzymatic activity. J. Biol. Chem..

[41] Wu X.S., Bao T.H., Ke Y., Sun D.Y., Shi Z.T., Tang H.R., Wang L. (2016). Hint1 suppresses migration and invasion of hepatocellular carcinoma cells in vitro by modulating girdin activity. Tumour. Biol..

[42] Kiseljak-Vassiliades K., Mills T.S., Zhang Y., Xu M., Lillehei K.O., Kleinschmidt-DeMasters B.K., Wierman M.E. (2017). Elucidating the role of the desmosome protein P53 apoptosis effector related to PMP-22 in growth hormone tumors. Endocrinology.

[43] Yang L., Chen Y., Cui T., Knösel T., Zhang Q., Albring K.F., Huber O., Petersen I. (2012). Desmoplakin acts as a tumor suppressor by inhibition of the Wnt/β-catenin signaling pathway in human lung cancer. Carcinogenesis.

[44] Ding Y., Lv C., Zhou Y., Zhang H., Zhao L., Xu Y., Fan X. (2021). Vimentin loss promotes cancer proliferation through up-regulating Rictor/AKT/β-catenin signaling pathway. Exp. Cell Res..

[45] Wang L., Mohanasundaram P., Lindström M., Asghar M.N., Sultana G., Misiorek J.O., Jiu Y., Chen H., Chen Z., Toivola D.M. (2022). Vimentin suppresses inflammation and tumorigenesis in the mouse intestine. Front. Cell. Dev. Biol..

[46] Lodhi G., Kim Y.S., Hwang J.W., Kim S.K., Jeon Y.J., Je J.Y., Ahn C.B., Moon S.H., Jeon B.T., Park P.J. (2014). Chitooligosaccharide and its derivatives: Preparation and biological applications. Biomed. Res. Int..

[47] Zhai X., Li C., Ren D., Wang J., Ma C., Abd El-Aty A.M. (2021). The impact of chitooligosaccharides and their derivatives on the In Vitro and In Vivo antitumor activity: A comprehensive review. Carbohydr. Polym..

[48] Park J.K., Chung M.J., Choi H.N., Park Y.I. (2011). Effects of the molecular weight and the degree of deacetylation of chitosan oligosaccharides on antitumor activity. Int. J. Mol. Sci..

[49] Zou P., Yang X., Zhang Y., Du P., Yuan S., Yang D., Wang J. (2016). Antitumor effects of orally and intraperitoneally administered chitosan oligosaccharides (COSs) on S180-bearing/residual mouse. J. Food Sci..

[50] Ashrafizadeh M., Zarrabi A., Mirzaei S., Hashemi F., Samarghandian S., Zabolian A., Hushmandi K., Ang H.L., Sethi G., Kumar A.P. (2021). Gallic acid for cancer therapy: Molecular mechanisms and boosting efficacy by nanoscopical delivery. Food Chem. Toxicol..

[51] Wang R., Ma L., Weng D., Yao J., Liu X., Jin F. (2016). Gallic acid induces apoptosis and enhances the anticancer effects of cisplatin in human small cell lung cancer H446 cell line via the ROS-dependent mitochondrial apoptotic pathway. Oncol. Rep..

[52] Khorsandi K., Kianmehr Z., Hosseinmardi Z., Hosseinzadeh R. (2020). Anti-cancer effect of gallic acid in presence of low level laser irradiation: ROS production and induction of apoptosis and ferroptosis. Cancer Cell Int..

[53] Salah R., Michaud P., Mati F., Harrat Z., Lounici H., Abdi N., Drouiche N., Mameri N. (2013). Anticancer activity of chemically prepared shrimp low molecular weight chitin evaluation with the human monocyte leukaemia cell line, THP-1. Int. J. Biol. Macromol..

[54] Böckelmann L.C., Felix T., Calabrò S., Schumacher U. (2021). YKL-40 protein expression in human tumor samples and human tumor cell line xenografts: Implications for its use in tumor models. Cell Oncol..

[55] Shao R. (2013). YKL-40 acts as an angiogenic factor to promote tumor angiogenesis. Front. Physiol..

[56] Eldaly M.N., Metwally F.M., Shousha W.G., EL-Saiid A.S., Ramadan S.S. (2020). Clinical potentials of MiR-576-3p, MiR-613, NDRG2 and YKL40 in colorectal cancer patients. Asian Pac. J. Cancer Prev..

[57] Povea-Cabello S., Oropesa-Ávila M., de la Cruz-Ojeda P., Villanueva-Paz M., de la Mata M., Suárez-Rivero J.M., Álvarez-Córdoba M., Villalón-García I., Cotán D., Ybot-González P. (2017). Dynamic reorganization of the cytoskeleton during apoptosis: The two coffins hypothesis. Int. J. Mol. Sci..

[58] Pradelli L.A., Villa E., Zunino B., Marchetti S., Ricci J.E. (2014). Glucose metabolism is inhibited by caspases upon the induction of apoptosis. Cell Death Dis..

[59] Lu K., Rui G., Liu F., Yang L., Deng X., Shi S., Li Q. (2018). 14-3-3ε is a nuclear matrix protein, and its altered expression and localization are associated with curcumin-induced apoptosis of MG-63 cells. Oncol. Lett..

[60] Weaver V.M., Carson C.E., Walker P.R., Chaly N., Lach B., Raymond Y., Brown D.L., Sikorska M. (1996). Degradation of nuclear matrix and DNA cleavage in apoptotic thymocytes. J. Cell. Sci..

[61] Yan C., Duanmu X., Zeng L., Liu B., Song Z. (2019). Mitochondrial DNA: Distribution, mutations, and elimination. Cells.

[62] Zhao H., Ming T., Tang S., Ren S., Yang H., Liu M., Tao Q., Xu H. (2022). Wnt signaling in colorectal cancer: Pathogenic role and therapeutic target. Mol. Cancer.

[63] Ho I.Y.M., Abdul Aziz A., Mat Junit S. (2020). Evaluation of anti-proliferative effects of *Barringtonia racemosa* and gallic acid on Caco-2 cells. Sci. Rep..

